# From Molecular Pathogenesis to Therapeutic Innovations in Human Cytomegalovirus Research

**DOI:** 10.3390/pathogens15090997

**Published:** 2026-09-21

**Authors:** Alexia Damour, Mathilde Larribau, Gaëtan Ligat

**Affiliations:** 1 Université Limoges, Inserm, CHU Limoges, UMR 1092, Limoges, France; 2 Univ Toulouse, INSERM, CNRS, Infinity - Toulouse Institute for Infectious and Inflammatory Diseases, Toulouse, France

Human cytomegalovirus (HCMV) remains one of the most clinically significant human pathogens, representing a major cause of congenital infection worldwide and a serious threat to immunocompromised individuals, including transplant recipients and HIV/AIDS patients. Increasing evidence also points to a potential role of HCMV in glioblastoma (GB), the most aggressive and lethal primary malignant brain tumor in adults, although the mechanisms underlying this association and the precise role of HCMV in GB remain to be fully elucidated. One important consideration is that current anti-HCMV therapies remain insufficient. Although several antiviral agents are available, their use is limited in certain patient populations due to significant toxicity. Moreover, the emergence of antiviral resistance represents an additional major challenge, particularly with the main currently available therapeutic options. Despite decades of intensive research, HCMV continues to challenge both fundamental virology and clinical management due to its remarkable biological complexity, lifelong persistence, and sophisticated interactions with the host immune system.

This Special Issue of *Pathogens*, entitled “Human Cytomegalovirus: From Molecular Pathogenesis to Therapeutic Innovations”, was conceived to provide an updated and integrated perspective on recent advances in HCMV biology, pathogenesis, and therapeutic development. The six contributions assembled in this issue reflect the breadth of contemporary HCMV research, spanning molecular mechanisms of infection, virus–host interactions, immune responses, tissue-specific disease manifestations, and emerging antiviral strategies.

A central theme emerging from this collection is the extraordinary capacity of HCMV to manipulate host cellular pathways to establish productive infection, persistence, and disease. Viral proteins and chemokine receptor homologues play critical roles in modulating cellular signaling and immune responses. In particular, recent work examining the viral G protein-coupled receptor pUS27 highlights the increasingly recognized contribution of HCMV-encoded receptor homologues to viral pathogenesis and immune evasion, illustrating the sophisticated mechanisms through which HCMV alters host-cell physiology [1]. These findings reinforce the concept that HCMV should be viewed not merely as an opportunistic pathogen but rather as a highly adapted regulator of cellular and immune functions.

Another important aspect covered in this Special Issue concerns the interaction between HCMV and the host immune system. The characterization of antigen-specific T-cell responses and cytokine profiles in transplant recipients provides further insight into the complexity of immune control of HCMV in clinically vulnerable populations. Such studies highlight the importance of understanding not only viral replication but also the quality, magnitude, and functional characteristics of antiviral immunity in determining clinical outcomes [2]. Together, these observations emphasize the need for increasingly integrated approaches combining virology, immunology, and clinical investigation.

The clinical diversity of HCMV infection is also illustrated by its ability to affect distinct tissues and contribute to disease beyond the traditionally recognized manifestations in immunocompromised patients. HCMV infection of the anterior segment of the eye has increasingly been recognized as an important cause of corneal endotheliitis and secondary glaucoma, including in immunocompetent individuals/patients [3]. This work highlights the complex interplay among viral persistence, local immune regulation, inflammation, and tissue damage, emphasizing the importance of tissue-specific mechanisms in HCMV pathogenesis.

HCMV may also contribute to chronic disease through persistent immune activation and interactions with other pathological conditions. In people living with HIV, HCMV seropositivity and immune responses to HCMV have been associated with inflammatory processes and cardiovascular disease. The study by Waters et al. investigates the potential value of HCMV-encoded interleukin-10 (cmvIL-10) in predicting coronary artery disease in people living with HIV, providing new insights into the potential contribution of viral immunomodulatory proteins to long-term comorbidities [4]. These findings further illustrate how the consequences of HCMV infection may extend beyond acute viral disease and involve complex interactions among viral persistence, immune dysregulation, and chronic inflammation.

The potential involvement of HCMV in GB represents another emerging area of considerable interest. The contribution by Flores et al. provides an overview of the current evidence linking HCMV with GB biology and discusses the potential therapeutic implications of targeting the virus in this highly aggressive malignancy [5]. The proposed mechanisms include modulation of oncogenic signaling, immune evasion, cellular metabolism, angiogenesis, and tumor-cell invasiveness. Although the precise role of HCMV in GB requires further validation, these observations illustrate how the study of virus–tumor interactions may open new avenues for therapeutic intervention.

Therapeutically, this Special Issue underscores both the progress achieved and the limitations that remain. The identification of new antiviral targets and compounds is therefore a major priority. In this context, the study of a potent HCMV inhibitor with post-entry activity specifically in differentiating myelo-monocytic cells provides an example of how a better understanding of cell-type-specific viral biology can inform the development of next-generation antiviral strategies [6]. Such approaches may complement existing polymerase-, kinase-, and terminase-targeting therapies and help overcome some of the limitations associated with current treatments.

Importantly, HCMV research increasingly benefits from technological innovations, including transcriptomic and single-cell approaches, advanced imaging, CRISPR-based functional genomics, and increasingly sophisticated cellular and tissue models. These approaches are transforming our ability to dissect viral replication, persistence, host responses, and tissue-specific pathogenesis at unprecedented resolution. Their continued development is likely to accelerate the identification of novel therapeutic targets and biomarkers and to facilitate the translation of fundamental discoveries into clinically relevant applications.

Collectively, the works presented in this Special Issue illustrate the multidimensional nature of HCMV research and the necessity of integrating molecular virology, immunology, and clinical investigation. From viral receptor biology and immune responses to tissue-specific disease, glioblastoma, and antiviral development, the contributions assembled here highlight both the remarkable adaptability of HCMV and the diversity of challenges that remain to be addressed. They also remind us that significant questions remain, particularly regarding the mechanisms governing tissue-specific pathogenesis, the determinants of inter-individual variability in immune control, the biological significance of viral persistence, and the long-term consequences of infection. Addressing these questions will require continued collaboration across disciplines and the development of experimental models that more faithfully reproduce the complexity of HCMV infection in vivo.

We hope that this Special Issue will serve as a valuable resource for researchers, clinicians, and students working in virology, infectious diseases, immunology, oncology, and translational medicine. By bringing together complementary perspectives on HCMV biology and therapeutic development, we aim to contribute to a deeper understanding of this complex pathogen and to stimulate the development of innovative strategies to better prevent, diagnose, and treat HCMV-associated diseases.
